# The evolution and impact of sarcopenia in severe aplastic anaemia survivors following allogeneic haematopoietic cell transplantation

**DOI:** 10.1002/jcsm.13449

**Published:** 2024-03-25

**Authors:** Dandan Chen, Zhaohu Yuan, Yuan Guo, Weifeng Liu, Zixuan Cheng, Lihua Ye, Wenjian Mo, Xinhua Wei

**Affiliations:** ^1^ Department of Radiology, Guangzhou First People's Hospital, School of Medicine South China University of Technology Guangzhou China; ^2^ Department of Blood Transfusion, Guangzhou First People's Hospital, School of Medicine South China University of Technology Guangzhou China; ^3^ Guangzhou First People's Hospital, School of Medicine South China University of Technology Guangzhou China; ^4^ Department of Hematology, Guangzhou First People's Hospital, School of Medicine South China University of Technology Guangzhou China

**Keywords:** Haematopoietic cell transplantation, Prognostic, Nomogram, Sarcopenia, Severe aplastic anaemia

## Abstract

**Background:**

Sarcopenia is a potential risk factor for adverse outcomes in haematopoietic cell transplantation (HSCT) recipients. We aimed to explore longitudinal body changes in muscle and adipose mass and their prognostic value in allogeneic HSCT‐treated severe aplastic anaemia (SAA) patients.

**Methods:**

We retrospectively analysed consecutive SAA patients who underwent allogeneic HSCT between January 2017 and March 2022. Measurements of pectoral muscle and corresponding subcutaneous fat mass were obtained via chest computed tomography at baseline and at 1 month, 3 months, 6 months, and 12 months following HSCT. Sarcopenia was defined as pectoral muscle index (PMI) lower than the sex‐specific median at baseline. Changes in body composition over time were evaluated by generalized estimating equations. Cox regression models were used to investigate prognostic factors affecting overall survival (OS) and failure‐free survival (FFS). A nomogram was constructed from the Cox regression model for OS.

**Results:**

We included 298 adult SAA patients (including 129 females and 169 males) with a median age of 31 years [interquartile range (IQR), 24–39 years] at baseline. Sarcopenia was present in 148 (148/298, 50%) patients at baseline, 218 (218/285, 76%) patients post‐1 month, 209 (209/262, 80%) patients post‐3 month, 169 (169/218, 78%) patients post‐6 month, and 129 (129/181, 71%) patients post‐12 month. A significant decrease in pectoral muscle mass was observed in SAA patients from the time of transplant to 1 year after HSCT, and the greatest reduction occurred in post 1–3 months (*P* < 0.001). The sarcopenia group exhibited significantly lower 5‐year OS (90.6% vs. 100%, log‐rank *P* = 0.039) and 5‐year FFS (89.2% vs. 100%, log‐rank *P* = 0.021) than the nonsarcopenia group at baseline. Sarcopenia at baseline (hazard ratio, HR, 6.344; 95% confidence interval, CI: 1.570–25.538; *P* = 0.01; and HR, 3.275; 95% CI: 1.159–9.252; *P* = 0.025, respectively) and the delta value of the PMI at 6 months post‐transplantation (ΔPMI6) (HR, 0.531; 95% CI: 0.374–0.756; *P* < 0.001; and HR, 0.666; 95% CI: 0.505–0.879; *P* = 0.004, respectively) were demonstrated to be independent prognostic factors for OS and FFS in SAA patients undergoing HSCT, and were used to construct the nomogram. The C‐index of the nomogram was 0.75, and the calibration plot showed good agreement between the predictions made by the nomogram and actual observations.

**Conclusions:**

Sarcopenia persists in SAA patients from the time of transplant to the 1‐year follow‐up after HSCT. Both sarcopenia at baseline and at 6 months following HSCT are associated with poor clinical outcomes, especially in patients with persistent muscle mass loss up to 6 months after transplantation.

## Introduction

Severe aplastic anaemia (SAA), a rare nonmalignant haematologic disorder, was first described in the late 19th century by the German pathologist Paul Ehrlich.^1^ It is characterized by peripheral pancytopenia, bone marrow (BM) hypocellularity, and haematopoietic stem cell niche number impairment and is associated with considerable morbidity and mortality.^2^ Allogeneic haematopoietic stem cell transplantation (HSCT) from HLA‐matched sibling donors (MSDs) is the preferred first‐line therapy for SAA patients younger than 40 years.^3^, ^4^, ^5^ With recent advances in supportive care, conditioning regimens and posttransplant immunosuppression, overall survival (OS) after HSCT approaches 70–90%, which means that the importance of managing survivors is increasing.^6^ In practice, allogeneic HSCT‐associated complications and medical treatments often cause tremendous changes in body composition, especially through disrupted muscle metabolism.^7^ Furthermore, body composition parameters are associated with long‐term health outcomes, which makes treatment‐related body composition changes an important issue for HSCT survivorship.^8^, ^9^ However, less attention has been given to body weight recovery in the posthospital phase, and there is no consensus among healthcare professionals about the best policy to promote recovery. Therefore, it is important to study the natural history of the related body composition complication for better understanding and insight to minimizing the impact on patient‐related outcomes.

Currently, the importance of identifying specific body composition changes during HSCT has emerged as an issue of interest. Sarcopenia, defined as a loss of skeletal muscle mass, has received attention as a sensitive early marker of treatment effectiveness in oncology^10^, ^11^, ^12^ as well as in haematological diseases.^13^, ^14^, ^15^ A few studies have shown that sarcopenia at baseline is associated with poor clinical outcomes after transplantation, which mostly focused on changes in body composition prior to transplantation.^16^, ^17^ In our previous study of 123 SAA patients treated with HSCT,^18^ we also found that sarcopenia, defined by chest computed tomography (CT) data obtained prior to transplantation, was an independent risk factor for decreased 3‐year OS and 3‐year fail‐free survival (FFS). However, the longitudinal changes in body composition during HSCT treatment in patients with haematologic disease, especially the subtle changes over time in SAA patients, which may play an integral role in guiding survivorship care planning and subsequent lifestyle interventions, are still unknown.

Although few studies^8^, ^9^, ^19^ have reported longitudinal data on nutritional status or weight loss in survivors of HSCT with different haematologic malignancies in recent years, nutritional parameters are usually assessed by body mass index (BMI), anthropometric data, or body weight‐for‐age *z* scores. Unfortunately, they cannot reliably distinguish which weight loss was the result of muscle loss or which weight gain was the result of fat deposition during treatment. Currently, CT is widely used to noninvasively quantify skeletal muscle and adipose tissue, which is used for other purposes to explore the cross‐sectional area of muscles and adipose depots captured in the field of view as surrogate markers.^20^, ^21^ Analyses of the evolution of body composition during different treatments by CT imaging have been performed for various diseases.^22^, ^23^, ^24^ In the clinical setting as part of standard clinical care to help exclude transplant‐related complications such as pulmonary infection, pulmonary oedema, and graft‐versus‐host disease (GVHD), SAA patients routinely receive chest CT scans during transplantation that can be used opportunistically to assess skeletal muscle and adipose tissue without additional radiation exposure or cost.

Although chest CT is not an imaging modality recommended by the revised European Working Group on Sarcopenia in Older People for the definition and diagnosis of sarcopenia,^25^ additional research has suggested that the pectoral muscle area (PMA) obtained opportunistically by chest CT is a validated proxy for total body fat‐free mass and is associated with the prognosis of several diseases.^18^, ^21^, ^26^, ^27^ Therefore, the first aim of this study was to describe the longitudinal body composition changes in terms of muscle and adipose tissue areas examined by chest CT from baseline to 1 year after transplantation in SAA patients. Furthermore, to deepen the understanding and develop future preventive and interventional approaches, we also explored the impact of body composition changes on patient prognosis at 1 year post‐transplantation.

## Methods

### Patient selection and data definitions

**Figure 1:**
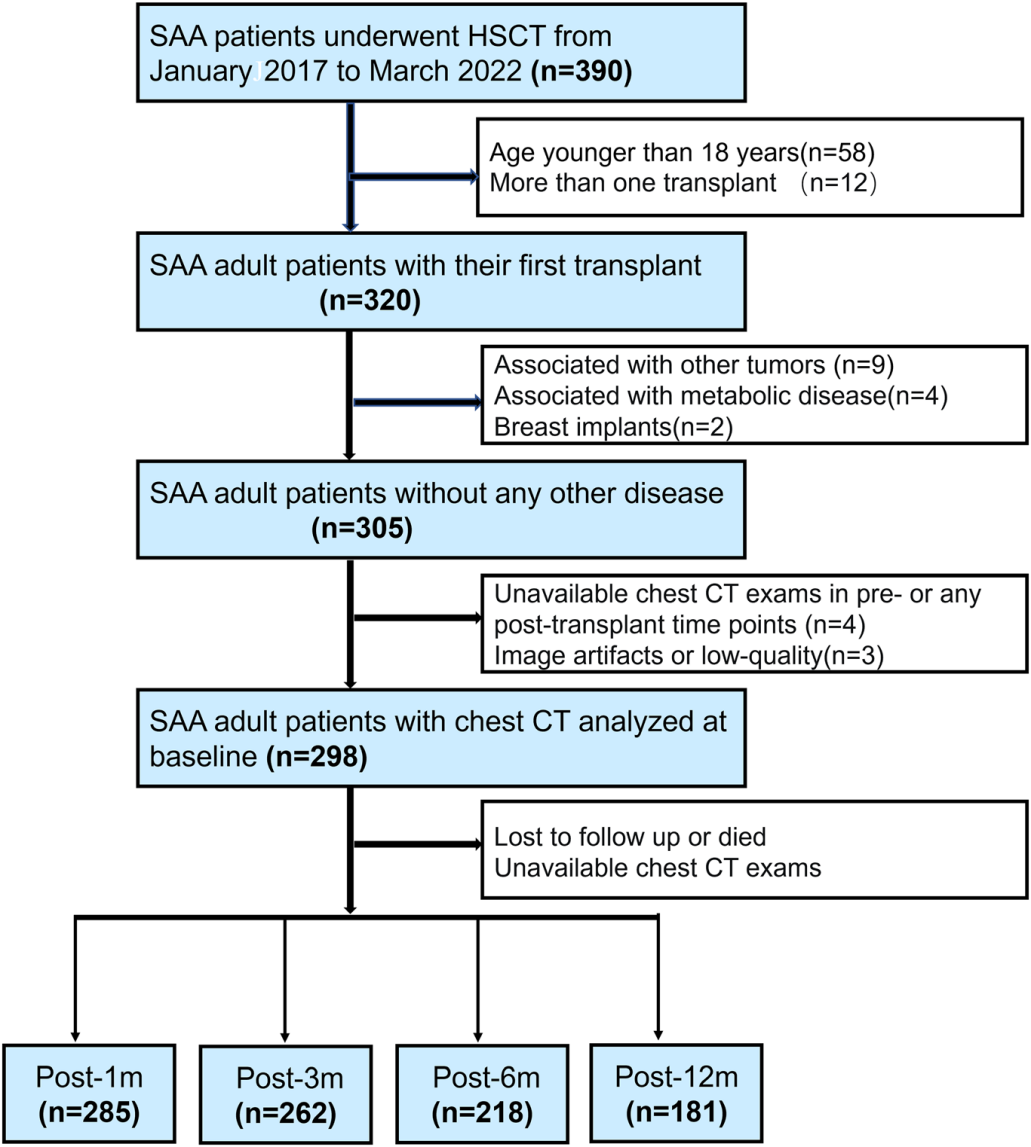
Flow diagram of the participants in the study. AA, severe aplastic anaemia; HSCT, haematopoietic cell transplantation.

This retrospective study was approved by the institutional review board of our institution, and the requirement for written informed consent was waived. Between January 2017 and March 2022, we retrospectively reviewed 390 consecutive SAA patients who underwent allogeneic HSCT from the medical records database. The inclusion and exclusion criteria for this study are shown in Figure 1. A total of 298 patients at baseline were ultimately enrolled in this study. The follow‐up data were updated on 21 December 2022.

Patient characteristics at baseline were collected from electronic medical records and included demographic characteristics (age at HSCT, sex, and BMI), clinical characteristics (diagnosis, sex of donor to recipient, donor type, conditioning regimen, and stem cell source), and laboratory examination (serum albumin and total protein). The clinical characteristics and laboratory examination results were defined according to previously methods.^18^

### Computed tomography imaging protocol

Noncontrast‐enhanced CT images were acquired with a 320 multirow detector CT scanner (Aquilion ONE; Toshiba Medical Systems, Tokyo, Japan) from the thoracic inlet to the diaphragm. The imaging protocol was as follows: tube voltage, 120 kV; automatic tube current, 10–300 mA; rotation time, 0.5 s; and slice thickness, 5 mm.

### Pectoralis muscle and adipose tissue assessment

**Figure 2:**
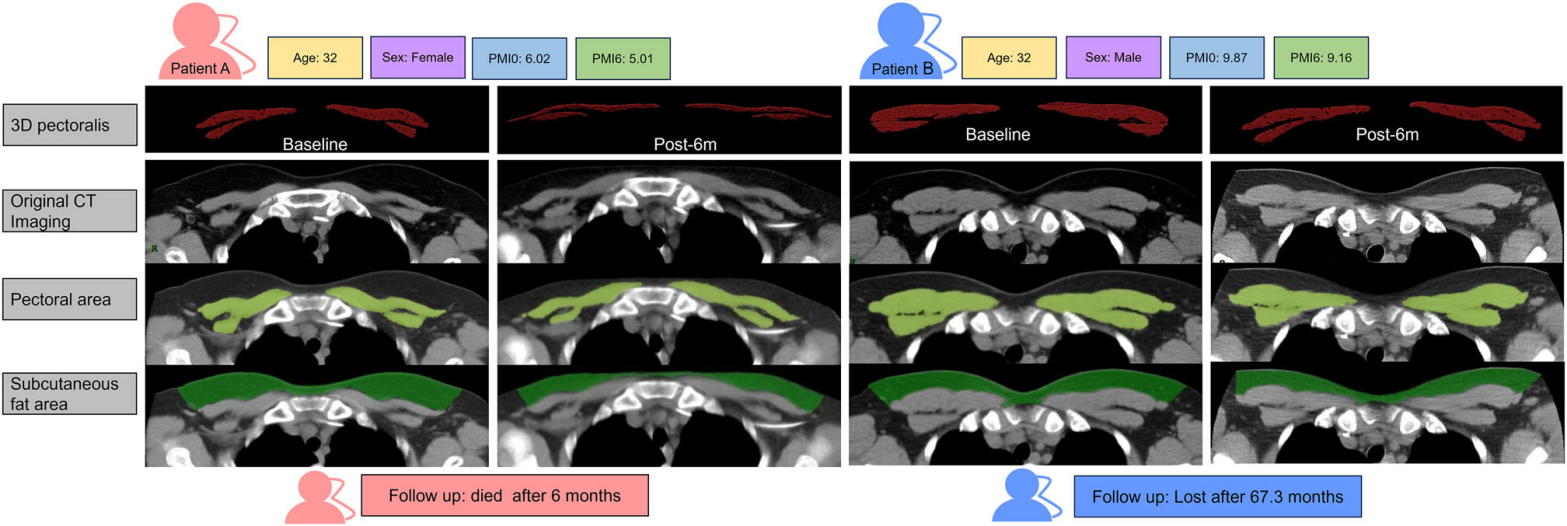
On axial unenhanced chest CT images, changes in the pectoral muscle area (CT thresholds ranging from −29 to 150 HU, green) and fat mass area (CT thresholds ranging from −190 to −30 HU, bottle green) were observed in sections above the aortic arch in SAA patients pre‐ and post‐ 6 months after HSCT. Patient A: A 32‐year‐old female with sarcopenia (PMI = 6.02 cm^2^/m^2^) at baseline, and a decreased PMI of 5.01 cm^2^/m^2^ after 6 months of HSCT; the patient died after 6 months of follow‐up. Patient B: A 32‐year‐old male with nonsarcopenia (PMI = 9.87 cm^2^/m^2^) at baseline and remaining nonsarcopenia with slight muscle loss post‐6 months (PMI = 9.16 cm^2^/m^2^) was included; the patient was lost to follow‐up after 67.3 months.

Chest CT imaging was assessed at baseline (within 1 month before transplantation) and at post‐1 month (1 month ± 30 days), post‐3 months (3 months ± 30 days), post‐6 months (6 months ± 30 days), and post‐12 months (12 months ± 30 days) relative to HSCT treatment, respectively. The quantitative parameters were measured by semiautomated software (Syngo Volume tool, Siemens Healthcare, Germany) and included the average area of bilateral PMA (including major and minor) (cm^2^), pectoral muscle density (PMD) (HU), the corresponding subcutaneous fat area (SFA) (cm^2^), and subcutaneous fat density (SFD) (HU) (Figure 2). Images were analysed by a single trained observer (radiologist with 10 years of clinical experience) blinded to the clinical outcome. The reliability of these measured markers was evaluated after reassessing 100 randomly selected CT images at baseline by the same observer (after 4 weeks of washout, intraobserver) and a second observer (radiologist with 5 years of clinical experience, interobserver).

The pectoral muscle index (PMI) and subcutaneous fat index (SFI) were calculated for each patient at each time point using the following formulas^28^: PMI = PMA in centimetres squared (cm^2^)/height squared in meters squared (m^2^), and SFI=SFA in centimetres squared (cm^2^)/height squared in meters squared (m^2^). As in our previous study,^18^ the sex‐specific median value of the PMI at baseline was selected as the cut‐off for classifying patients into sarcopenia (lower than the sex‐specific median PMI at baseline) and nonsarcopenia (higher than the sex‐specific median PMI at baseline) groups both at baseline and post‐6 month, respectively.

The delta value of the PMI (ΔPMI) was defined as the difference between the PMI parameters measured at each follow‐up time point post‐HSCT and the baseline measurements. The calculation formula was as follows: ΔPMIn = PMIn‐PMI0 (*n* = 1 m, 3 m, 6 m, 12 m, PMI0 and PMIn represent the PMI values at baseline and at the follow‐up time point of month post‐HSCT, respectively).

### Follow‐up and transplant‐related outcomes

SAA patients were routinely followed up at 1, 3, 6, and 12 months after transplantation, and at their discretion, clinicians screened patients for lung disease and transplant‐related complications based on condition. OS was defined as the time from the date of HSCT to the date of death from any cause or the last follow‐up. FFS was defined as survival with response, excluding primary or secondary graft failure, relapse, and death.

### Statistical analyses

Normally distributed continuous variables are expressed as the mean ± standard deviation (SD), and nonnormally distributed continuous variables are expressed as the median and interquartile range (IQR). Categorical variables are presented as counts (percentages). Intraobserver and interobserver reliabilities of the measurement parameters were assessed by reporting the intraclass correlation coefficient (ICC) and 95% confidence interval (CI), and Bland–Altman analysis was conducted to evaluate bias (mean difference). Comparisons of demographic characteristics, clinical characteristics, and laboratory examinations between sarcopenia and nonsarcopenia groups at baseline were performed using the independent *t* test or Mann–Whitney *U* test for continuous data and the *χ* ^2^ test, Fisher exact test or Kruskal–Wallis H test for categorical variables. Body composition (including the PMA, PMI, SFA, and SFI) changes pre‐ and post‐CT scans (post‐1 month, post‐3 months, post‐6 months, and post‐12 months) for each patient were evaluated by generalized estimating equation models, and Bonferroni correction was used to account for multiple testing.

Kaplan–Meier curves were used to assess the cumulative rates of OS and FFS between sarcopenia and nonsarcopenia groups both at baseline and post‐6 months, respectively. Cox proportional hazards regression analyses were performed to evaluate the independent prognostic factors for OS and FFS. Prognostic factors with *P* < 0.05 in the univariate competitive risk Cox regression analysis were included in the multivariate analysis to determine hazard ratios (HRs) and 95% CIs. A nomogram was developed from the final model to visualize the prognostic value of each risk factor for predicting the 1‐, 3‐, and 5‐year OS rates. The predictive accuracy of the nomogram was evaluated by discrimination and calibration. Discrimination was quantified by the concordance index (C‐index) with its respective 95% CI. Statistical analyses were performed with SPSS software (ver. 21.0; SPSS, Inc., Chicago, IL, USA) and R software (R Foundation for Statistical Computing, Vienna, Austria). Two‐sided *P* values <0.05 were considered indicative of statistical significance.

## Results

### Patient characteristics at baseline

A total of 298 adult SAA patients (including 129 females and 169 males) with a median age of 31 years [interquartile range (IQR), 24–39 years] at baseline were included. According to the above definition criteria for sarcopenia, a PMI < 6.57 cm^2^/m^2^ in females and a PMI < 8.82 cm^2^/m^2^ in males were defined as sarcopenia. In total, 148 patients (63 females and 85 males, mean ages of 31.7 ± 0.9 years) were categorized as sarcopenia group, while 150 patients (66 females and 84 males, mean ages of 33.2 ± 0.8 years) were classified as the nonsarcopenia group at baseline, respectively. Seventy‐one (71/298, 24%) patients were older than 40 years (including 33 patients with sarcopenia and 38 patients without sarcopenia), with the oldest age being 59 years. Compared with those in the nonsarcopenic group, sarcopenia patients had significantly lower BMI, PMA, PMI, SFA, and SFI at baseline (*P* < 0.001). The baseline characteristics of the patients in the study cohort are listed in Table 1.

**Table 1:** Comparison of SAA patients' characteristics between sarcopenia and non‐sarcopenia at baseline

Characteristics	Sarcopenia (*n* = 148)	Non‐sarcopenia (*n* = 150)	*P* value
Demographic characteristics			
Age (years)	29 (23–37.5)	33 (25–40)	0.092
Age, *n* (%)			0.538
18–40 years	115 (78)	112 (75)	
≥40 years	33 (22)	38 (25)	
Sex, *n* (%)			0.803
Female	63 (43)	66 (44)	
Male	85 (57)	84 (56)	
BMI (kg/m^2^)	20.70 (14.50–29.40)	22.05 (16.40–39.50)	**<0.001**
Clinical characteristics			
Diagnosis, *n* (%)			0.460
SAA	109 (74)	116 (77)	
vSAA	39 (26)	34 (23)	
Sex of donor to recipient, *n* (%)			0.960
Male to male	58 (39)	56 (37)	
Male to female	48 (32)	53 (35)	
Female to male	25 (17)	25 (17)	
Female to female	17 (12)	16 (11)	
Donor type, *n* (%)			0.812
MSD	50 (34)	49 (33)	
MUD	43 (29)	40 (27)	
HID	55 (37)	61 (40)	
Conditioning regimen, *n* (%)			0.228
CY + BU + ATG	74 (50)	62 (41)	
FLU + CY + ATG	34 (23)	33 (22)	
TBI + CY + ATG + FLU	32 (22)	48 (32)	
Others	8 (5)	7 (5)	
Stem cell source, *n* (%)			0.741
BM + PB	105 (71)	109 (73)	
PB	43 (29)	41 (27)	
Laboratory examination			
Alb (g/L)	36.16 ± 0.41	36.79 ± 0.32	0.973
TP (g/L)	63.43 ± 0.57	63.44 ± 0.50	0.152
Radiographic characteristics			
PMA0	17.55 (13.88–21.75)	25.46 (18.48–29.83)	**<0.001**
PMI0	6.36 (5.37–7.68)	9.24 (7.53–10.56)	**<0.001**
PMD0	41.26 ± 0.89	44.66 ± 0.95	0.414
SFA0	9.00 (5.19–15.14)	11.83 (8.13–17.46)	**<0.001**
SFI0	3.33 (1.77–5.64)	4.58 (2.94–6.59)	**<0.001**
SFD0	−91.77 ± 1.02	−93.00 ± 0.89	0.505

The bold values were statistically significant.

Alb, albumin; ATG, antithymocyte immunoglobulin; BM, bone marrow; BU, busulfan; CY, cyclophosphamide; FLU, fludarabine; HID, haploidentical donor; MSD, matched sibling donor; MUD, matched unrelated donor; PB, peripheral blood; PMA, pectoralis muscle area; PMD, pectoralis muscle density; PMI, pectoralis muscle index; SAA, severe aplastic anaemia; SFA, subcutaneous fat area; SFD, subcutaneous fat density; SFI, subcutaneous fat index; TBI, total body irradiation; TP, total protein; vSAA, very SAA.

### Reliability of the measurements

All the measurements of the PMA, PMD, SFA, and SFD showed excellent interobserver and intraobserver reliability (ICC = 0.890–0.973, *P* < 0.001; Table S1), and the Bland–Altman analysis of the measured parameters is shown in Figure S1.

### Longitudinal changes in body composition

**Figure 3:**
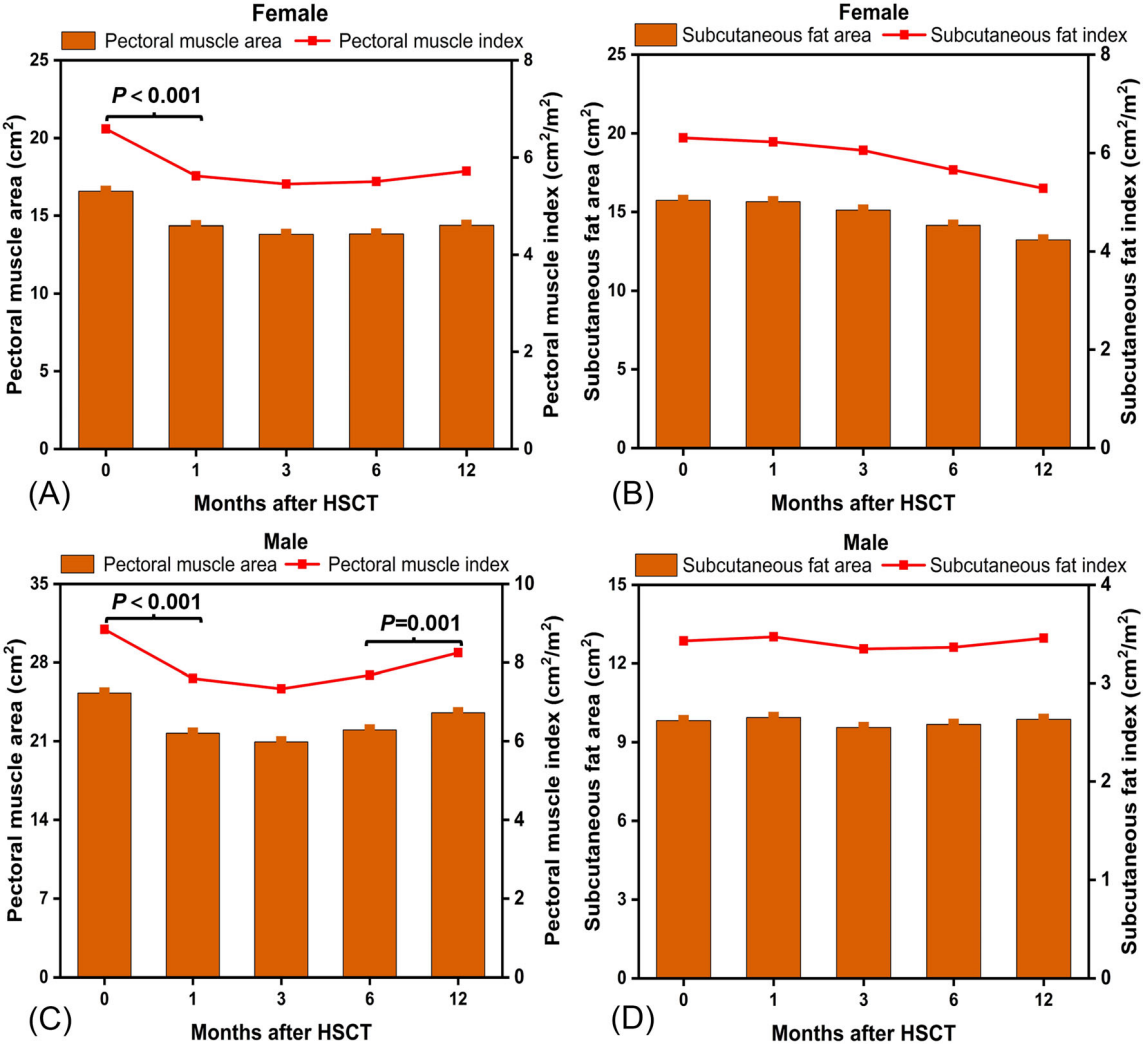
Time‐course changes in the average area (graphs) and index (line graphs) of pectoral muscle and fat mass in SAA patients at the 1‐year follow‐up after HSCT. (A) Mean PMA and PMI in females; (B) mean SFA and SFI in females; (C) mean PMA and PMI in males; (D) mean SFA and SFI in males. PMA, pectoralis muscle area; PMI, pectoralis muscle index; SFA, subcutaneous fat area; SFA, subcutaneous fat index.

Measurements of chest CT imaging (PMA, ΔPMA, PMI, ΔPMI, and SFA, ΔSFA, SFI, ΔSFI) at each time point for different sexes are shown in Table 2. The average PMA and PMI were higher in males than in females at each time point (*P* < 0.001), but the SFA and SFI were lower (*P* < 0.001). Skeletal muscle (PMA and PMI) decreased significantly during the 1‐year follow‐up after HSCT in both females and males (*P* < 0.001) (Figure 3A,C). However, there was no significant change in fat mass (SFA or SFI) in either sex (*P* > 0.05) (Figure 3B,D).

**Table 2:** Radiographic characteristics at different time points within 1‐year follow‐up after HSCT

Variable	Baseline (*n* = 298)		Post‐1 month (*n* = 285)		Post‐3 months (*n* = 262)		Post‐6 months (*n* = 218)		Post‐12 months (*n* = 181)	
Sarcopenia, *n* (%)	148 (50%)		218 (76%)		209 (80%)		169 (78%)		129 (71%)	
Sex	F (*n* = 129)	M (*n* = 169)	F (*n* = 123)	M (*n* = 162)	F (*n* = 117)	M (*n* = 145)	F (*n* = 92)	M (*n* = 126)	F (*n* = 78)	M (*n* = 103)
PMA (cm^2^)	16.57 ± 0.38	25.30 ± 0.49	14.36 ± 0.27	21.72 ± 0.39	13.82 ± 0.32	20.94 ± 0.42	13.84 ± 0.32	22.02 ± 0.49	14.38 ± 0.34	23.54 ± 0.49
ΔPMA (cm^2^)			−2.31 ± 0.30	−3.68 ± 0.36	−2.90 ± 0.37	−4.48 ± 0.42	−2.78 ± 0.42	−3.24 ± 0.48	−1.69 ± 0.39	−1.70 ± 0.41
PMI (cm^2^/m^2^)	6.52 ± 0.16	8.84 ± 0.17	5.62 ± 0.12	7.60 ± 0.13	5.40 ± 0.13	7.33 ± 0.15	5.41 ± 0.14	7.68 ± 0.17	5.64 ± 0.15	8.25 ± 0.18
ΔPMI (cm^2^/m^2^)			−0.91 ± 0.12	−1.28 ± 0.13	−1.13 ± 0.15	−1.58 ± 0.15	−1.11 ± 0.17	−1.13 ± 0.17	−0.64 ± 0.15	−0.60 ± 0.14
SFA (cm^2^)	15.73 ± 0.86	9.83 ± 0.52	15.65 ± 0.72	9.94 ± 0.47	15.11 ± 0.75	9.56 ± 0.51	14.15 ± 0.64	9.68 ± 0.62	13.23 ± 0.77	9.87 ± 0.75
ΔSFA (cm^2^)			−0.16 ± 0.49	−0.06 ± 0.22	−0.59 ± 0.61	−0.34 ± 0.31	−0.74 ± 0.55	−0.18 ± 0.38	−0.88 ± o.78	0.04 ± 0.48
SFI (cm^2^/m^2^)	6.26 ± 0.36	3.43 ± 0.18	6.23 ± 0.31	3.47 ± 0.16	5.99 ± 0.31	3.35 ± 0.17	5.57 ± 0.26	3.36 ± 0.21	5.20 ± 0.32	3.46 ± 0.25
ΔSFI (cm^2^/m^2^)			−0.06 ± 0.20	−0.02 ± 0.08	−0.25 ± 0.25	−0.12 ± 0.11	−0.33 ± 0.22	−0.07 ± 0.13	−0.35 ± 0.31	0.02 ± 0.17

Δ, the difference between each time point after HSCT and the baseline (ΔPMI = PMIn − PMI0, *n* = 1 month, 3 months, 6 months, 12 months); HSCT, haematopoietic stem cell transplantation; PMA, pectoralis muscle area; PMD, pectoralis muscle density; PMI, pectoralis muscle index; SFA, subcutaneous fat area; SFD, subcutaneous fat density; SFI, subcutaneous fat index.

In the whole cohort of female patients, both the PMA and PMI showed a significant downward trend during the 1‐year follow‐up after transplantation (*P* < 0.001). A major reduction in skeletal muscle was observed at post‐1 to 3 months (*P* < 0.001), and did not cease until post‐12 months, but the deterioration was somewhat alleviated after post‐3 months (*P* > 0.05) (Figure 3A). Regarding male patients, in addition to the significant recovery of skeletal muscle mass during the post‐6 to 12 months span (*P* = 0.001), there was a similar downward trend in the PMA and PMI as in females (Figure 3C). Additionally, compared to the nonsarcopenia group, the sarcopenia group exhibited lower skeletal muscle (PMA and PMI) and fat mass (SFA and SFI) at each time point after HSCT. Similar variation in these measured parameters were observed in both the sarcopenia and nonsarcopenia group during the 1‐year follow up (Figure S2).

### Survival analysis

**Figure 4:**
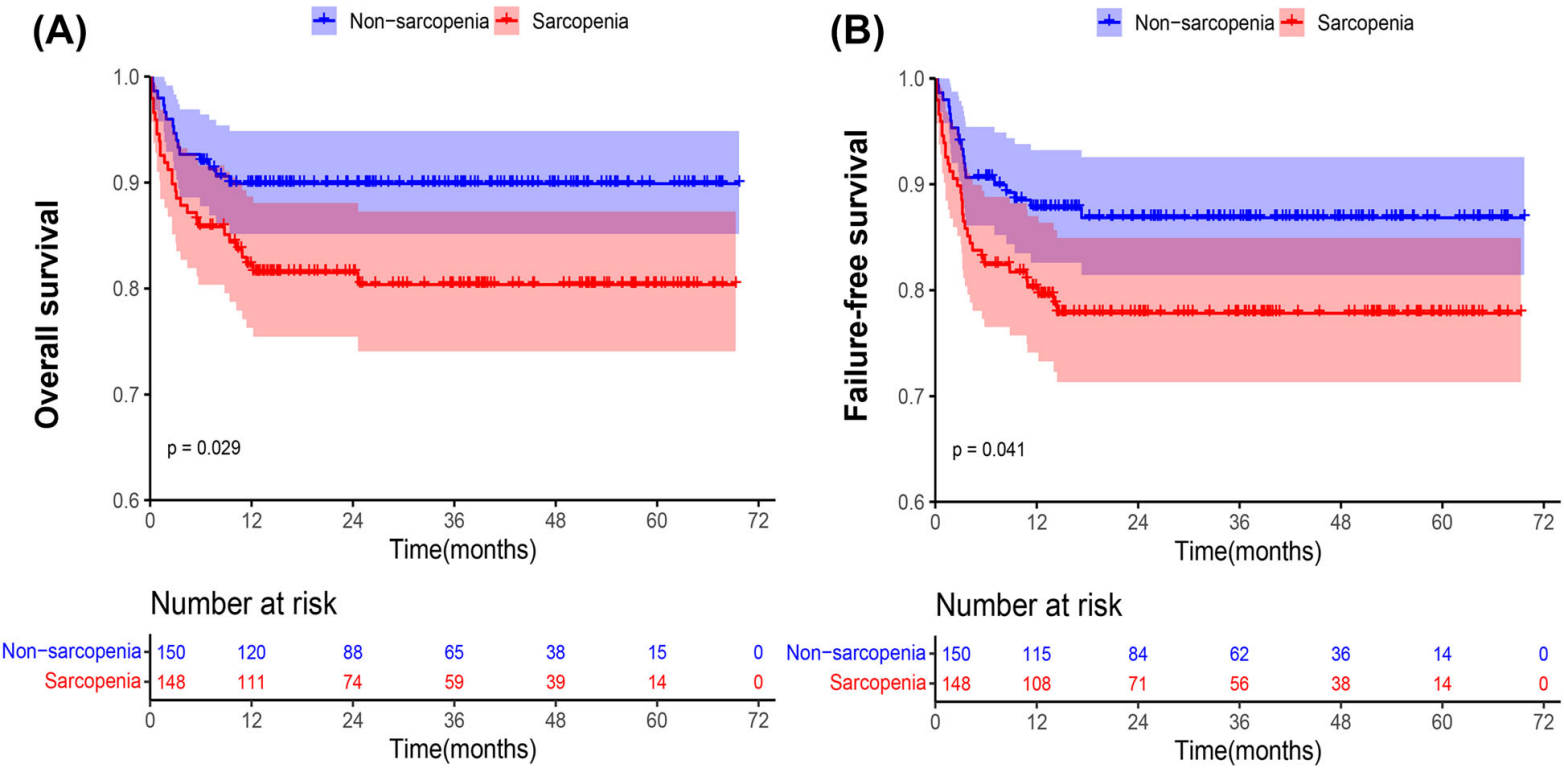
Kaplan–Meier curves for (A) OS and (B) FFS of SAA patients with sarcopenia (red) and without sarcopenia (blue) at baseline.

At a median follow‐up of 26.4 months (range, 0.1–69.7 months), 43 (43/298, 14%) patients (including 28 patients with sarcopenia and 15 patients without sarcopenia) died, with an average time of 4.2 months (range, 0.1–24.6 months). The 1‐year, 3‐year and 5‐year OS and FFS rates were 82.2%, 80.4%, and 80.4% and 79.5%, 77.8%, and 77.8%, respectively, in the sarcopenia group. Similarly, the 1‐year, 3‐year and 5‐year OS rates were the same as 89.9% and the FFS rates were 87.8%, 86.8%, and 86.8%, respectively, in the nonsarcopenia group. Compared to the nonsarcopenia group, the sarcopenia group at baseline had a lower 5‐year OS (80.4% vs. 89.9%, log‐rank *P* = 0.029) and 5‐year FFS (77.8% vs. 86.8%, log‐rank *P* = 0.041) (Figure 4A,B).

In univariate analysis, age, sex, BMI, diagnosis, donor type, conditioning regimen, stem cell source, albumin, total protein, baseline SFI, baseline sarcopenia, ΔPMI1, ΔPMI3, ΔPMI6, and ΔPMI12 were included as candidate variables in the multivariate model. According to the final multivariate analysis, both baseline sarcopenia (HR, 6.344; 95% CI: 1.570–25.538; *P* = 0.01 and HR, 3.275; 95% CI: 1.159–9.252; *P* = 0.025, respectively) and ΔPMI6 (HR, 0.531; 95% CI: 0.374–0.756; *P* < 0.001 and HR, 0.666; 95% CI: 0.505–0.879; *P* = 0.004, respectively) were independent prognostic factors for OS and FFS (Table 3).

**Table 3:** Univariate and multivariate analyses for OS and FFS.

Characteristics	OS				FFS			
	Univariable		Multivariable		Univariable		Multivariable	
	HR (95% CI)	*P*	HR (95% CI)	*P*	HR (95% CI)	*P*	HR (95% CI)	*P*
Age (years)								
18–40	Ref				Ref		Ref	
≥40	0.616 (0.274–1.385)	0.241			0.674 (0.328–1.385)	0.283		
Sex								
F	Ref				Ref			
M	1.319 (0.711–2.448)	0.380			1.114 (0.638–1.946)	0.704		
BMI (kg/m^2^)								
≥18.5	Ref				Ref			
<18.5	1.455 (0.675–3.136)	0.339			0.729 (0.355–1.498)	0.390		
Diagnosis								
SAA	Ref				Ref			
vSAA	1.591 (0.841–3.012)	0.153			1.232 (0.666–2.279)	0.506		
Donor type								
MSD	Ref		Ref		Ref			
MUD	1.186 (0.471–2.988)	0.717	2.541 (0.276–23.417)	0.411	0.841 (0.374–1.894)	0.676		
HID	2.523 (1.178–5.408)	0.017	5.710 (0.699–46.662)	0.104	1.727 (0.906–3.294)	0.097		
Stem cell source								
PB	Ref				Ref			
BM + PB	1.542 (0.740–3.215)	0.248			1.674 (0.838–3.341)	1.144		
Conditioning regimen								
CY + BU + ATG	Ref				Ref			
FLU + CY + ATG	0.781 (0.346–1.763)	0.551			1.083 (0.539–2.176)	0.824		
TBI + CY + ATG + FLU	0.996 (0.490–2.024)	0.990			1.067 (0.549–2.075)	0.847		
Others	1.011 (0.236–4.326)	0.988			0.902 (0.212–3.837)	0.889		
Alb (g/L)								
≥34	Ref				Ref			
<34	1.090 (0.543–2.190)	0.809			1.441 (0.788–2.636)	0.235		
TP (g/L)								
≥60	Ref				Ref			
<60	1.114 (0.555–2.238)	0.762			1.325 (0.718–2.447)	0.368		
Radiographic analysis at baseline								
Sarcopenia (<median PMI)	1.980 (1.057–3.707)	0.033	6.344 (1.570–25.638)	**0.010**	1.793 (1.016–3.163)	0.044	3.275 (1.159–9.252)	**0.025**
SFI (<median SFI)	1.068 (0.587–1.942)	0.829			1.056 (0.610–1.828)	0.847		
Radiographic analysis in post‐transplant								
ΔPMI1	0.883 (0.695–1.122)	0.308			0.877 (0.702–1.095)	0.246		
ΔPMI3	0.898 (0.716–1.126)	0.352			0.943 (0.769–1.156)	0.573		
ΔPMI6	0.643 (0.488–0.848)	0.002	0.531 (0.374–0.756)	**<0.001**	0.768 (0.601–0.981)	0.034	0.666 (0.505–0.879)	**0.004**
ΔPMI12	1.756 (0.958–3.219)	0.069			1.373 (0.911–2.070)	0.130		

The bold values were statistically significant.

Δ, the difference between each time point after HSCT and the baseline (ΔPMI = PMIn − PMI0, n = 1 month, 3 months, 6 months, 12 months); SAA, severe aplastic anaemia; vSAA, very SAA; MSD, matched sibling donor; MUD, matched unrelated donor; HID, haploidentical donor; CY, cyclophosphamide; BU, busulfan; ATG, antithymocyte immunoglobulin; FLU, fludarabine; TBI, total body irradiation; BM, bone marrow; PB, peripheral blood; Alb, albumin; TP, total protein; PMA, pectoralis muscle area; PMI, pectoralis muscle index; PMD, pectoralis muscle density; SFA, subcutaneous fat area; SFI, subcutaneous fat index; SFD, subcutaneous fat density.

### Association between sarcopenia and prognosis at the post‐6 months time point

**Figure 5:**
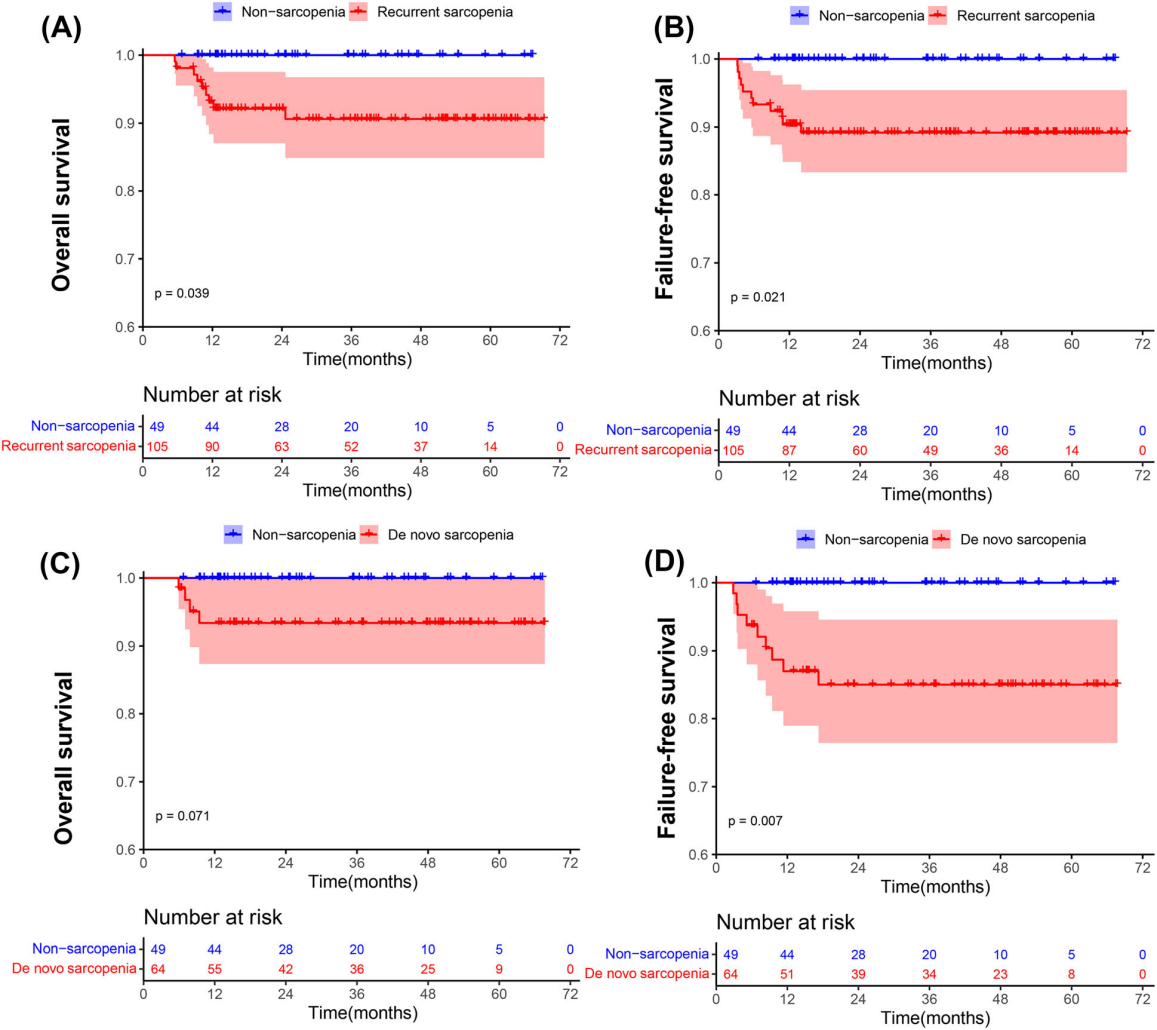
Kaplan–Meier curves for (A) OS and (B) FFS of SAA patients with recurrent sarcopenia (red) and nonsarcopenia (blue), and (C) OS and (D) FFS of SAA patients with de novo sarcopenia (red) and nonsarcopenia (blue) at 6 months after HSCT.

During the follow‐up of 6 months after HSCT, the data of 218 patients (169 patients with sarcopenia and 49 patients without sarcopenia) were reviewed. A total of 105 (105/218, 48%) individuals were diagnosed with sarcopenia both at baseline and post‐6 months and were defined as the recurrent sarcopenia group. The de novo sarcopenia group included 64 (64/218, 29%) patients who developed sarcopenia after 6 months without sarcopenia at baseline. Of the remaining 49 (49/218, 22%) patients without sarcopenia, only five patients experienced resolution of sarcopenia following transplantation. According to Kaplan–Meier analysis, compared with those in the nonsarcopenia group, the recurrent sarcopenia group exhibited lower cumulative OS (90.6% vs. 100%, log‐rank *P* = 0.039; Figure 5A) and FFS (89.2% vs. 100%, log‐rank *P* = 0.021; Figure 5B). Although there was no significant difference in OS between the de novo sarcopenia group and the nonsarcopenia group (de novo sarcopenia group vs. nonsarcopenia group, 93.4% vs. 100%, log‐rank *P* = 0.071; Figure 5C), the FFS was lower in the de novo sarcopenia group (de novo sarcopenia group vs. nonsarcopenia group, 85.0% vs. 100%, log‐rank *P* = 0.007; Figure 5D).

### Prognostic nomogram for overall survival

**Figure 6:**
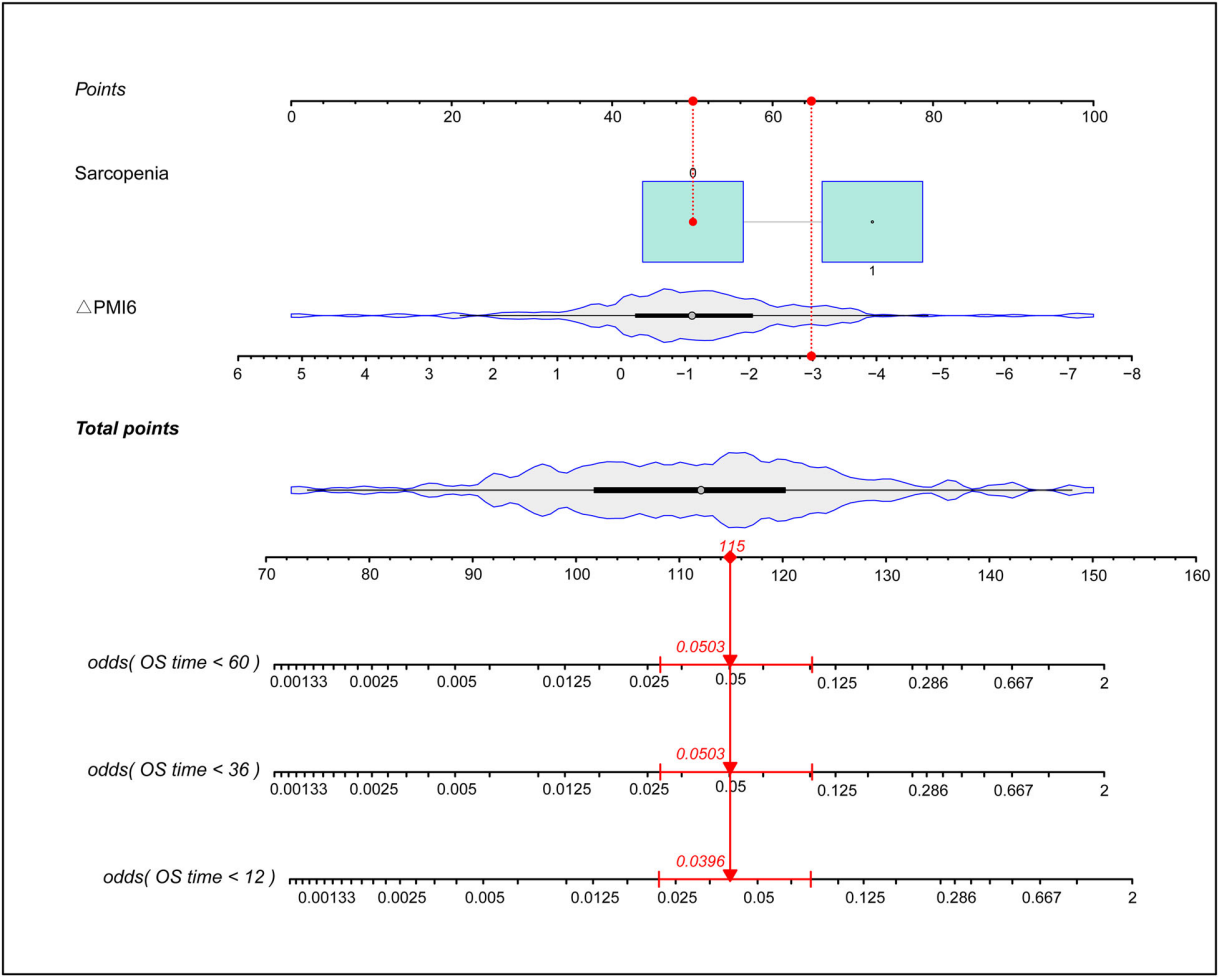
Nomogram for predicting 1‐, 3‐ and 5‐ year OS.

A nomogram was constructed to predict OS by using two significant prognostic factors (sarcopenia at baseline and the ΔPMI6), which were determined by Cox regression analysis, and the 3‐ and 5‐year OS were the same (Figure 6). Sarcopenia at baseline was the largest contributor to the variance in the nomogram. The C‐index of the nomogram was 0.75 (95% CI: 0.579–0.924), which was higher than that of sarcopenia at baseline (0.59, 95% CI: 0.686–7.235). The calibration plot for the probability of 1‐, 3‐, and 5‐year OS showed that the nomogram results were linearly related to the actual observations (Figure S3).

### Discussion

In our study, we evaluated the longitudinal changes in skeletal muscle mass (PMA and PMI) and subcutaneous fat mass (SFA and SFI) derived from chest CT in SAA patients in the first year after HSCT. First, female patients had lower skeletal muscle and higher subcutaneous fat mass than male patients. Both the PMA and PMI decreased from the time of transplant evaluation until 1 year after HSCT, and the major reduction occurred at 1–3 months. Only a very few patients with sarcopenia demonstrated improvement after transplantation, and up to 30% of patients developed de novo sarcopenia within 6 months following HSCT. Second, sarcopenia at baseline was shown to be an independent risk factor for OS and FFS as in our previous studies. Moreover, ΔPMI6 was demonstrated to be an independent protective factor for SAA survivor patients after HSCT. A higher ∆PMI6 indicates a greater PMI and improved sarcopenia after 6 months of follow‐up, which was associated with superior clinical outcomes.

SAA was one of the first diseases to benefit from allogeneic HSCT, given that successful engraftment with absence meant a cure for most patients.^29^ However, allogeneic HSCT is a complex procedure that can cause many complications ranging from GVHD to treatment‐ or progression‐related mortality and may often cause marked changes in body composition.^7^ Historically, age has been considered a crucial factor in transplant outcomes, with the age of 40 years being considered the limiting factor for selecting allogeneic HSCT as the first‐line treatment for SAA patients.^6^ In our study, 71 patients (71/298, 24%) were older than 40 years, with the oldest age being 59 years. Similar to the findings in the study of older adults undergoing HSCT by Koch et al.,^30^ we also observed no significant association between age and the risk of death in allogeneic HSCT patients. Based on the current data, we speculate that chronological age may not be serve as an exclusion criterion in isolation as a limiting factor for transplantation. Instead, a comprehensive assessment of muscle mass, strength, and physical performance, collectively known as sarcopenia, should be considered when determining the eligibility of older or otherwise medically unfit patients for HSCT.^31^

In our study, with an increase in the incidence of sarcopenia of 21–30%, the number of SAA patients who developed sarcopenia increased significantly during the 1‐year follow‐up after HSCT, and major muscle loss occurred from 1 to 3 months after transplantation. Muscle loss during early post‐transplantation was mainly due to severe anorexia and acute GVHD, both of which are major complications for survivors of allogeneic HSCT.^7^ Furthermore, the use of corticosteroids and immunosuppressive drugs for the treatment of acute GVHD may negatively affect muscle metabolism after HSCT.^32^ Moreover, lower body weight and muscle loss accounted for a greater incidence of GVHD following allogeneic HSCT. It is possible that poor nutritional status at the time of transplant influences inflammatory cytokine production, which, together with the conditioning regimen, could worsen gastrointestinal tract damage, favouring the development of acute GVHD.^9^, ^17^ Therefore, prevention and treatment of acute GVHD through the alleviation of sarcopenia must be prioritized. In addition, slight recovery of skeletal muscle mass was found from 6 to 12 months after HSCT, especially in males. There are several potential explanations for the differences. Firstly, as girls experience more rapid brain growth than boys during early childhood, female brains may be more susceptible to HSCT preconditioning, potentially resulting in neurocognitive impairment and inactivity.^19^, ^33^ Another study involving adult survivors of childhood acute lymphoblastic leukaemia also indicated that girls were less likely to meet the physical activity recommendations set by the Centers for Disease Control and Prevention compared to boys.^34^ Additionally, physical inactivity may cause imbalances between myokine production and muscle contraction fostering a pro‐inflammatory state and metabolic dysfunction that contribute to muscle loss.^35^

Compared to the findings of other similar studies, the trends in body composition in SAA patients following HSCT in our study were similar to those of other haematological diseases in previous studies, but more importantly, we had some new findings. In a retrospective study of 315 lymphoma patients who underwent allogeneic HSCT (*n* = 97),^36^ body composition was measured at the level of the L3 vertebral body on abdominal CT. A substantial decrease in lean body mass and increased sarcopenia were observed in lymphoma patients at 1 year and 2.5 years post‐HSCT. The long‐term trend in skeletal muscle parameters observed in that study was consistent with that observed in our study. Nevertheless, the subtle changes in body composition within the first year after transplantation were ignored although this period was supposed to be the most important for SAA survivors. In our study, we found that the most important stage for the transplant recipients of SAA patients was the first year after HSCT, and the survival rate of the survivors basically stabilized thereafter. Xiao et al.^37^ also demonstrated that the muscle area initially decreased during treatment and returned to baseline during the 24‐month follow‐up in lymphoma patients undergoing chemotherapy. In our study, the muscle area of SAA patients also showed a slow recovery at 6–12 months after the obvious decrease at 1–3 months post‐HSCT, especially in male patients, but it remained lower than baseline at the 1‐year follow‐up after HSCT.

Sarcopenia is a frequent complication in patients treated with HSCT because of long‐term chemotherapy or immunosuppressants, and it is an increasingly recognized independent risk factor associated with poor outcomes in patients undergoing HSCT.^16^, ^17^, ^38^, ^39^ As shown in other studies of haematologic malignancies, we demonstrated that baseline sarcopenia in SAA patients was a poor independent prognostic factor for OS and FFS. The cumulative survival of sarcopenia patients treated with HSCT for SAA was significantly lower than that of nonsarcopenia patients. This finding was consistent with our previous studies.^18^ Furthermore, in addition to sarcopenia at baseline, the change in skeletal muscle parameters, expressed as the ∆PMI at 6 months postoperatively, was also demonstrated to be independently correlated with OS and FFS. Further studies revealed that patients with recurrent sarcopenia in SAA patients at post‐6 months had significantly lower OS and FFS rates than those without sarcopenia. Previously, no other studies evaluated this point. Patients treated with HSCT who lost weight persistently for 3–6 months were regarded as severely malnourished.^8^ These findings suggest the importance of increasing skeletal muscle mass in improving OS and FFS in SAA survivors before transplantation and up to 6 months after transplantation. The mechanism by which sarcopenia contributes to lower OS and FFS data remains uncertain and is likely multifactorial. First, the disease burden (including disease status and co‐morbidities), the side effects of aggressive treatment and a variety of frequent and severe gastrointestinal problems related to GVHD‐related complications after transplantation, may result in loss of appetite, insufficient oral intake, and malnutrition in patients.^16^ Second, sarcopenia, in turn lead to decreased tolerance to cytotoxic drugs used for disease treatment, contributing to increased treatment‐related toxicity and further disrupting the treatment process. This vicious cycle may compromise patient tolerance and elevate susceptibility to infection, toxicity, and other complications, thereby increasing the risk of an inability to completely eradicate cancer cells, causing therapeutic resistance and subsequent relapse.^40^

A nomogram predicting the 1‐, 3‐, and 5‐year OS was constructed based on the final model of the Cox regression analysis, which included sarcopenia at baseline and ΔPMI6. A C‐index of 0.75 in the nomogram indicated excellent discrimination. As the nomogram improved conventional prognostication indicators by adding the patient's general physical condition before and after transplantation to well‐known prognostic factors, the nomogram may help stratify patients in clinical trials with HSCT to improve clinical outcomes.

Some limitations of this study should be noted. First, this study was designed retrospectively and involved a single centre. However, to our knowledge, this was the largest study of longitudinal and subtle changes in body composition in SAA patients after transplantation. Moreover, large‐scale single‐disease studies with strict inclusion and exclusion criteria tend to minimize data bias and systematic errors, improving the accuracy and reliability of the research results. Second, due to retrospective nature of the study, muscle strength and physical performance data were not collected, even though they are recommended as diagnostic criteria for sarcopenia.^41^ Third, as supportive care and nutritional interventions were nonstandardized and initiated as clinically needed, we did not have nutritional data and were unable to evaluate their impact on body composition in the study population. Prospective, controlled and multicentre studies are needed to ascertain the longitudinal changes in body composition after transplantation and their impact on clinical outcomes.

In conclusion, sarcopenia may emerge as a prevalent consequence of HSCT in SAA patients. Sarcopenia progresses through the transplant process and continues even after HSCT in most survivors during long‐term follow‐up. It remains important to actively maintain muscle mass in surviving patients with SAA not only at baseline with respect to HSCT but also during follow‐up, especially in those with persistent muscle mass loss up to 6 months after transplantation.

## Conflict of interest

The authors declare that there are no conflicts of interest.

## Funding

This study was supported by Guangdong Basic and Applied Basic Research Foundation (No. 2022A1515220019).

## Supporting information


**Figure S1.** Bland–Altman analysis of agreement for the measurement parameters of the two radiologists. The red and black horizontal dashed lines in each plot indicate the mean difference and 95% limits of agreement, respectively.

**Figure S2.** Time‐course changes in the average area (graphs) and index (line graphs) of pectoral muscle and fat mass in both sarcopenia and nonsarcopenia patients at the 1‐year follow‐up after HSCT. (A) mean PMA and PMI in females; (B) mean SFA and SFI in females; (C) mean PMA and PMI in males; (D) mean SFA and SFI in males.

**Figure S3.** Calibration plots for predicting 1‐, 3‐ and 5‐year OS.

**Table S1.** The reliability of measurement parameters of the two radiologists.
