# Supplementary figures and images for: The evolution and impact of sarcopenia in severe aplastic anaemia survivors following allogeneic haematopoietic cell transplantation
Source: J Cachexia Sarcopenia Muscle. 2024 Mar 25;15(3):1094–107. doi: 10.1002/jcsm.13449 (PMC11154763; doi:10.1002/jcsm.13449)

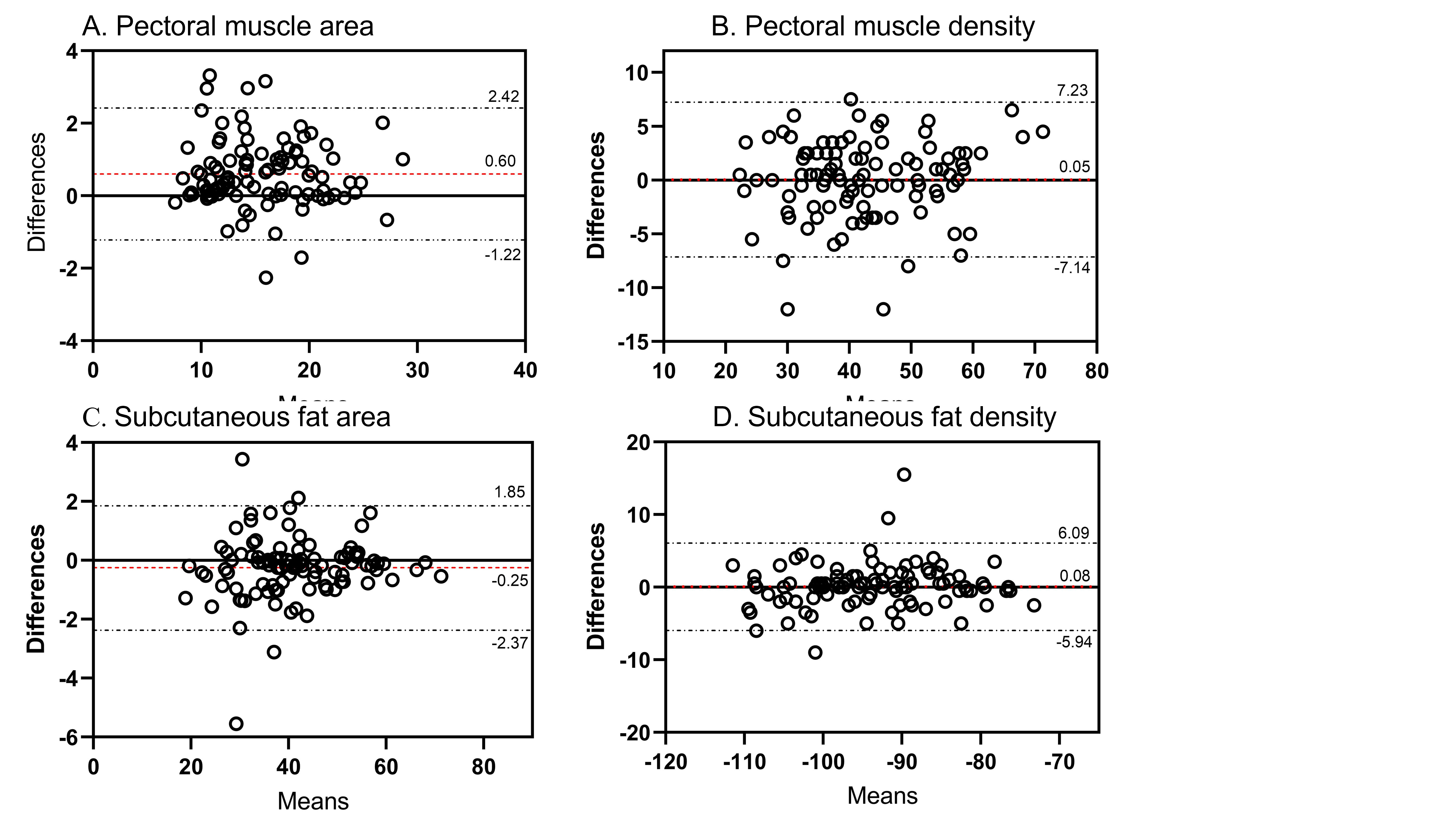

Supplement: Supplementary file 1 — Figure S1. Bland–Altman analysis of agreement for the measurement parameters of the two radiologists. The red and black horizontal dashed lines in each plot indicate the mean difference and 95% limits of agreement, respectively. [file JCSM-15-1094-s003.tif]

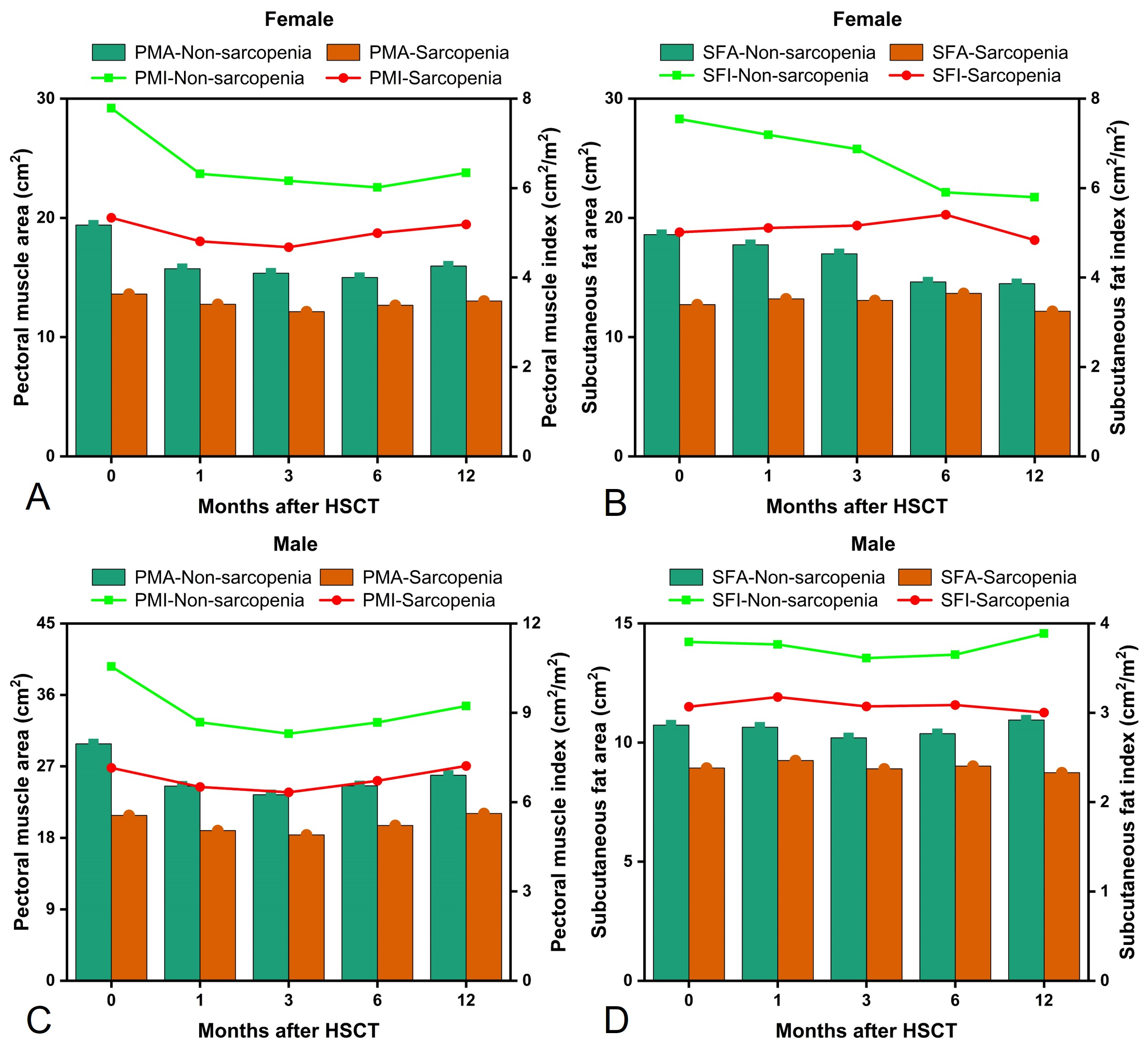

Supplement: Supplementary file 2 — Figure S2. Time‐course changes in the average area (graphs) and index (line graphs) of pectoral muscle and fat mass in both sarcopenia and nonsarcopenia patients at the 1‐year follow‐up after HSCT. (A) mean PMA and PMI in females; (B) mean SFA and SFI in females; (C) mean PMA and PMI in males; (D) mean SFA and SFI in males. [file JCSM-15-1094-s002.tif]

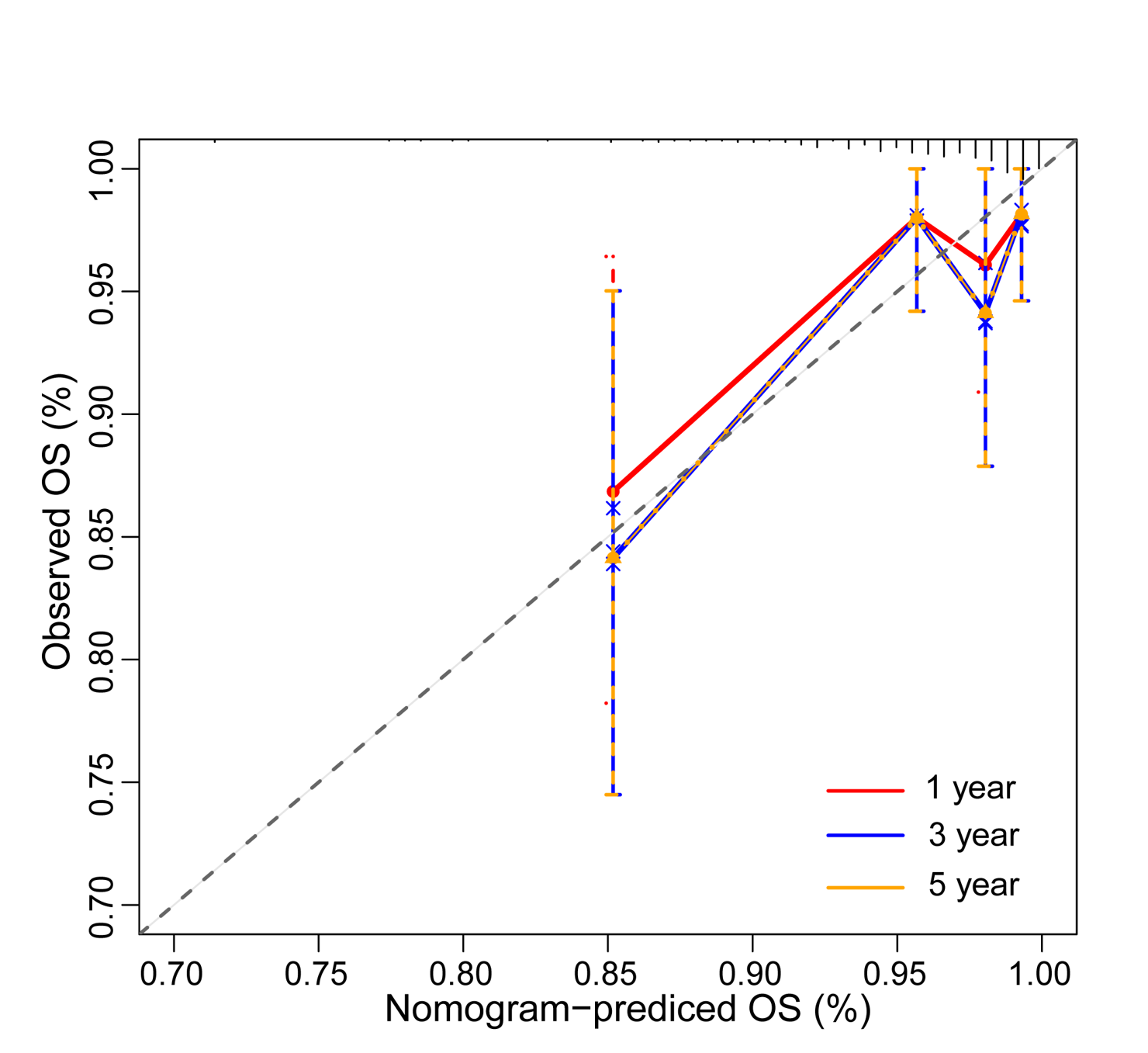

Supplement: Supplementary file 3 — Figure S3. Calibration plots for predicting 1‐, 3‐ and 5‐year OS. [file JCSM-15-1094-s004.tif]
