# Supplementary material for: The evolution and impact of sarcopenia in severe aplastic anaemia survivors following allogeneic haematopoietic cell transplantation
Source: J Cachexia Sarcopenia Muscle. 2024 Mar 25;15(3):1094–107. doi: 10.1002/jcsm.13449 (PMC11154763; doi:10.1002/jcsm.13449)
Supplement: Supplementary file 4 — Table S1. The reliability of measurement parameters of the two radiologists. [file JCSM-15-1094-s001.docx]

**Table S1.** The reliability of measurement parameters of the two radiologists

| **Characteristics** | **Reliability ICC (95%CI)** | | |
| --- | --- | --- | --- |
|  | **Intra-observer** |  | **Inter-observer** |
| **PMA** | 0.973 (0.922-0.987) |  | 0.910(0.716-0.966) |
| **PMD** | 0.944 (0.917-0.962) |  | 0.889(0.825-0.928) |
| **SFA** | 0.982 (0.972-0.988) |  | 0.949(0.835-0.977) |
| **SFD** | 0.948 (0.924-0.965) |  | 0.890(0.841-0.925) |

Abbreviations: ICC, Intraclass Correlation Coefficient; CI, Confidence Interval; PMA, pectoralis muscle area; PMD, pectoralis muscle density; SFA, subcutaneous fat area; SFD, subcutaneous fat density
